# The effects of surgically implanted dummy tags on the survival, growth performance, and physiology of pikeperch (*Sander lucioperca*)

**DOI:** 10.1007/s10695-017-0347-2

**Published:** 2017-02-03

**Authors:** Maciej Rożyński, Andrzej Kapusta, Krystyna Demska-Zakęś, Marek Hopko, Agnieszka Sikora, Zdzisław Zakęś

**Affiliations:** 1 Department of Aquaculture, The Stanisław Sakowicz Inland Fisheries Institute, Oczapowskiego 10, 10-719 Olsztyn, Poland; 2 Department of Hydrobiology, The Stanisław Sakowicz Inland Fisheries Institute, Oczapowskiego 10, 10-719 Olsztyn, Poland; 3 Department of Ichthyology, Faculty of Environmental Sciences, University of Warmia and Mazury, Oczapowskiego 5, 10-959 Olsztyn, Poland

**Keywords:** Blood biochemistry, Dummy transmitter, Tissue adhesive, Pikeperch, Radio telemetry, Suture

## Abstract

The aim of this work was to determine the impact of surgically implanted telemetry transmitters (TTs) on the growth, survival, hematological and biochemical indexes, and wound healing in juvenile pikeperch (*Sander lucioperca*) (body weight 60–90 g). Two incision suturing methods were used—silk sutures (experiment I—group ST) or tissue adhesive (experiment II—group GT). After tagging, the fish were held in a recirculating system for 35 days. No statistically significant differences were noted in the growth or condition indexes analyzed among the fish tagged with TT compared with those from the control groups (untagged). Substantial individual variability was noted, however, in the parameters examined in both the control and tagged groups. Among the hematological indexes, statistically significant differences were only noted in experiment I. Lower values of mean corpuscular volume and mean corpuscular hemoglobin were noted in group ST. Among the biochemical parameters, creatinine was statistically significantly threefold lower, magnesium and alkaline phosphatase (ALP) levels were lower, and ammonia levels were higher in group ST than in the control group. In experiment II, significant differences were only noted for ALP. Tissue adhesive was the superior and more effective method for closing the incision after TT implantation in juvenile pikeperch. This type of suturing facilitated faster healing, and it had less of an impact on juvenile pikeperch welfare.

## Introduction

Drastic, systematic declines in pikeperch (*Sander lucioperca*) catches from open waters have been observed since the early 1950s (FAO 2012, 2016). Consequently, restocking programs for this species have been implemented in several countries (e.g., Finland, Poland, and Iran) (Bartley and Rana 1998; Steenfeldt et al. 2015). These programs, as well as other research projects, have begun analyzing the effectiveness of restocking this species along with investigating little known aspects of pikeperch biology, such as spawning migrations (Koed et al. 2000; Koed et al. 2002). Essential data are acquired from observations of tagged fish. Until recently, primarily traditional methods of tagging pikeperch have been used, for example, hot branding, marking with dyes like alcian blue, or external and internal tags (Saura 1996; Hansson et al. 1997). Studies have also indicated that this species can be tagged successfully with coded wire tags (CWTs) or passive integrated transponders (PITs) (Zakęś and Hopko 2013; Zakęś et al. 2015).

One of the techniques that facilitate a better understanding and more effective monitoring of the various aspects of the lives of fish is telemetry (Baras and Lagardère 1995; Thorstad et al. 2013). Using telemetry transmitters (TTs) in ichthyological studies permits investigating the behavior and physiology of individuals in their natural environment; this method has become increasingly widespread recently, and it is developing continually (Thorstad et al. 2013). Frequently, telemetry study is the only reliable method that permits assessing the effectiveness of restocking, fish survival, spawning behavior, diel activity patterns, and habitat preferences (Cooke et al. 2013; Thorstad et al. 2013; Dudgeon et al. 2015).

Telemetry has also been used sporadically in studies of pikeperch with the aim of obtaining a better understanding of their biology and behavior, including spawning migratory routes. Reports of using telemetry transmitters in adult specimens (total body length (TL) >40 cm) of this species are found in the literature (Jepsen et al. 2000; Koed et al. 2002; Vehanen and Lahti 2003). Adult pikeperch are most frequently tagged with TTs that are inserted into the body cavity through a small incision, while the antenna is directed through an opening located just above it. The incision is closed with two or three silk sutures depending on the size of the incision and the size of the fish being tagged (Koed et al. 2000; Horký et al. 2006). Other methods of implantation incision closure, such as tissue adhesive or leaving the incision open, have been investigated in other fish species (Baras and Jeandrain 1998; Cooke et al. 2011).

The basic principle of tagging with this type of transmitter, as with other tagging methods, is that the presence of the transmitter in the fish body should not impact fish behavior, condition, or physiological or metabolic processes (Cooke et al. 2011). This is why as many factors as possible that could impact the welfare of the tagged fish during and after the procedure must be taken into consideration. For example, it is crucial to select the correct size tag for the size of the fish. Generally, the weight of the tag should not exceed 2% of that of the fish body (Winter 1996). It is also important to choose the appropriate implantation method (incision location and size, incision closure method, and the technique for directing the antenna outside of the body) (Mulcahy 2003; Cooke et al. 2011). Additionally, the experience of the personnel performing the procedure and the method for inducing general anesthesia also have a direct impact on the welfare of the fish (Mulcahy 2003). Developing optimal methods for tagging fish with telemetry transmitters will permit treating the data obtained from a group of tagged individuals as representative of the entire population (Bridger and Booth 2003; Cooke et al. 2011). One significant disadvantage of this tagging method is the lack of wider ranging, documented data and information on the impact telemetry transmitter implantation has on the health and physiological processes of the fish, and particularly on juveniles. This data could be provided through studies of hematological and blood biochemical indexes of the fish. Since these parameters are strongly correlated with external factors that impact the fish, as well as with various pathological changes, these parameters are often determined to assess fish health. For example, blood biochemical indexes (glucose, total protein, magnesium, etc.) are important biological tools in the assessment of stress, nutritional status, and water-mineral balance in fish (Brinn et al. 2012). Red blood cell indexes can be useful in diagnosing anemia. It should also be pointed out that erythrocytes are a general index of various physiological adaptation strategies associated with environmental changes (Val et al. 1992). Additionally, hematological tests can be useful in diagnosing many diseases, including infectious ones (e.g., IPN, IHN, or VHS) (Zorriehzahra et al. 2010) and in toxicological studies (Pereira et al. 2013). A certain degree of caution must be exercised when interpreting hematological results, because in fish, the generally accepted range of values for individual indexes often do not take into consideration factors such as fish sex, origin (natural environment or aquaculture), water quality, or season of the year. Even simple manipulations, such as catching and transporting or the blood sampling procedure, can impact the results of hematological and biochemical determinations (Clauss et al. 2008).

The aim of this work was to study the impact of two intraperitoneal telemetry implantation procedures, which differed in how the implantation incision was closed (silk or tissue adhesive sutures), on fish growth and survival and the hematological and biochemical parameters of juvenile pikeperch. The healing of the implantation incisions was also analyzed.

## Materials and methods

### Fish and initial rearing conditions

The study material was obtained through out-of-season spawning conducted at the Department of Sturgeon Breeding in Pieczarki (IFI Olsztyn, Poland) (Zakęś 2007). Larval and juvenile stages were reared in recirculating systems (RASs) in accordance with previously established procedures (Szkudlarek and Zakęś 2007). The fish with body weights of approximately 10 g were transported to the Department of Aquaculture (IFI Olsztyn, Poland) (transport time 2 h). Following the acclimatization phase, they were reared in experimental RAS outfitted with a rearing tank with a volume of 0.2 m^3^. The water temperature in the RAS was 20.7 ± 0.1 °C, the pH range was 7.70–8.05, and oxygenation at the water discharge from the rearing tank did not fall below 7.5 mg O_2_ l^−1^. The fish were reared until they had reached a body weight (BW) of about 60 g.

Two experiments were conducted. In experiment I, the fish standard length (SL) was 17.01 ± 0.52 cm (mean ± SD) and BW was 59.78 ± 4.55 g (mean ± SD). In experiment II, the fish used measured SL 19.67 ± 0.60 cm and BW 86.90 ± 6.43 g.

### Fish-tagging procedure

The day before the fish were tagged with TT, all of the pikeperch were tagged in the cheek with PITs (Fish Eagle, Lechlade, Great Britain) (material—bioglass, length—12.0 ± 0.4 mm, diameter—2.12 ± 0.07 mm, weight—93 mg) (Zakęś and Hopko 2013). This permitted identifying individual specimens. Before tagging, the fish were bathed in an aqueous solution of etomidate (Propiscin, IFI Olsztyn, Poland) at a concentration of 1.5 ml l^−1^ (Lambooij et al. 2009). After tagging with PITs and recovery time, the fish were returned to the RAS.

The fish were surgically implanted with radio transmitters (length 13 mm, diameter 5 mm, with a 21.5 cm external whip antenna; model F1515, ATS Inc., Isanti, MN, USA). The weight of the transmitters was 0.604–0.653 g (mean 0.63 g). The relative weight of the transmitters was <1.2% BW fish (experiment I) and <0.9% BW pikeperch (experiment II). In experiment I, non-absorbable surgical silk (Jedwab Polski Sp. z o.o., Milanówek, Poland) was used to make two sutures to close the implantation incision, while in experiment II, Surgibond tissue adhesive was used (SMI AG, St. Vith, Belgium). Before implantation, the pikeperch were anesthetized in an aqueous solution of etomidate (Propiscin) at a concentration of 1.5 ml l^−1^. After 3–4 min, the fish were in a state of general anesthesia that was apparent from the lack of balance and no reaction to external stimuli (Kristan et al. 2014). The transmitters were implanted to the body cavity through an incision measuring 10–15 mm that was made approximately 20 mm anterior to the pectoral fin, and the antenna was directed out of the body between the abdominal and anal fins (Wagner et al. 2011). After the procedure, the incision was disinfected with betadine (Lavipharm, Peania, Greece). When implanting TT and closing the incision with silk sutures, the procedure lasted 3–4 min and the recovery time ranged from 3 to 5 min. The length of the procedure of implanting and closing the incision with tissue adhesive was a mean of 1.5 min, and recovery time was 3–5 min. In experiment I, 18 fish (group ST) were tagged, and these fish were stocked into three rearing tanks (6 specimens per tank). The fish from the control group (18 specimens tagged only with PIT; group SC) were subjected to the same procedures as the experimental fish (with the exception of tagging and TT implantation) and were stocked into three tanks (6 specimens per tank). The same methodological principles were employed in experiment II (the group of fish tagged with TT were called group GT and the control group GC). After recovery in oxygenated containers with a volume of 0.08 m^3^, the fish were held in tanks with a volume of 0.2 m^3^. The specimens from a given tank/replicate were held in the same tank throughout the study period. The mean biomass of the stocks in the tanks in both experiments was 1.79 kg m^−3^ (experiment I) and 2.61 kg m^−3^ (experiment II). The fish were reared for 5 weeks in both experiments.

### Conditions proper of rearing and feeding fish

Water temperature (±0.1 °C) and oxygen concentration (±0.01 mg O_2_ l^−1^) at the rearing tank inflows and outflows were measured daily. The amounts of total ammonia nitrogen (TAN = NH_4_^+^-N + NH_3_-N; ±0.01 mg TAN l^−1^) and nitrites (NO_2_-N; ±0.01 mg NO_2_-N l^−1^) and pH (±0.01) were measured at the outflows of the rearing tanks weekly. The mean water temperature in both experiments was 22.0 ± 0.0 °C. In experiment I, the oxygen concentration at the tank outflows did not fall below 7.29 O_2_ l^−1^ (82.8% saturation), while in experiment II, it was 7.21 O_2_ l^−1^ (82.1% saturation). Concentrations of ammonia and nitrites at the outflows did not exceed 0.08 mg TAN l^−1^ and 0.007 mg NO_2_-N l^−1^ (experiment I) or 0.04 mg TAN l^−1^ and 0.001 mg NO_2_-N l^−1^ (experiment II). The water pH at the outflow in experiment I ranged from 7.96 to 8.00, while in experiment II, the range was 7.77–7.94.

In both experiments, the fish were fed the same feed—Aller Performa EX 3GR (AllerAqua, Christiansfeld, Denmark). The feed was delivered using an automatic band feeder (Fischtechnik GmbH, Nienburg, Germany) for 18 h d^−1^ (09:00–03:00). The daily feed ration was determined weekly at 1.5% of the fish biomass.

### Research procedures

The fish were measured and weighed individually just before implanting the TT (day 0—*d*_0_) (SL ± 0.1 cm; BW ± 0.01 g). Subsequent individual measurements were taken every 7 days (*d*_7_, *d*_14_, *d*_21_, *d*_28_, and *d*_35_). During measurements, each fish was identified with a PIT tag reader (Fish Eagle, Lechlade, Great Britain). During the individual measurements, the state of the silk sutures or tissue adhesive was assessed (Table 1; Deters et al. 2010), and the state of the abdominal incision site was examined macroscopically (Table 2; Miller et al. 2014). Throughout the experiment, the occurrence of redness, edema, inflammation, infection, and tissue necrosis at the implantation site was also monitored (Table 3).

**Table 1:** Silk or adhesive suture assessment criteria

Rank	Assessment criteria
0	Lack of silk/adhesive sutures
1	Silk/adhesive sutures partially cover incision
2	Silk/adhesive sutures fully cover incision

*Source*: Deters et al. (2010)

**Table 2:** Macroscopic assessment criteria of the incision site

Rank	Assessment criteria
0	Incision fully closed and healed/no trace of incision
1	Incision fully closed but not healed
2	Incision healing, sides of incision only partially connected with tissue
3	Incision healing, but sides of incision not closed/not connected with tissue
4	Less than 50% of wound open
5	More than 50% of wound open
6	Wound fully open

*Source*: Miller et al. (2014)

**Table 3:** Post-implantation incision assessment criteria

Rank	Assessment criteria
0	Clean incision
1	Some redness
2	Inflammation
3	Infection, necrosis

Feed consumption, expelled PIT or TT tags in the tanks, and fish behavior and mortality were monitored daily. The data collected served to calculate the following: daily growth rate—DGR (g d^−1^) = (BW_2_ − BW_1_) × *t* ^−1^, specific growth rate—SGR (% d^−1^) = 100 × (lnBW_2_ − lnBW_1_) × *t* ^−1^, Fulton’s condition coefficient—*F* = 100 × BW × SL^−3^, and feed conversion ratio—FCR = TFS × (FB − IB)^−1^, where BW_1_ is the initial fish body weight (g), BW_2_ is the final fish body weight (g), *t* is the rearing time (days), SL is the fish body length (cm), FB is the final stock biomass (g), IB is the initial stock biomass (g), and TFS is the total feed supply (g).

On the concluding days of experiments I and II (*d*_35_), approximately 1 ml of blood was drawn directly from the caudal vein of each specimen with a heparinized syringe (Sarstedt AG & Co., Nümbrecht, Germany) after the fish had been anesthetized (Propiscin, 1.5 ml l^−1^). The blood samples were used to determine the following hematological indexes: white blood cell count (WBC), red blood cell count (RBC), hemoglobin (HGB), hematocrit (HCT), and platelets (PLTs). The samples were also used to determine the following erythrocyte indexes: mean corpuscular volume (MCV), mean corpuscular hemoglobin (MCH), and mean corpuscular hemoglobin concentration (MCHC). Portions of the blood samples were centrifuged at a speed of 1500×*g* for 3 min (Fresco 17, Thermo Scientific, Waltham, USA). The material obtained was used to determine the following biochemical indexes: creatinine (CREA), total protein (TP), total bilirubin (BIL-T), alanine aminotransferase (ALT), alkaline phosphatase (ALP), calcium (Ca), albumin (ALB), globulin (GLB), glucose (GLU), magnesium (Mg), and ammonia (NH_3_). Hematological measurements were done with a BC-2800 VET semi-automatic hematology analyzer (Mindray, Shenzhen, China), while biochemical measurements were done with a BS-120 automatic chemistry analyzer (Mindray, Shenzhen, China).

### Statistical analysis

Statistical analysis was performed with Statistica 12 (StatSoft Inc., USA). The homogeneity of variance was tested using Levene’s test. The experiment results concerning growth data, silk and adhesive suture retention, and incision assessments were analyzed with repeated measure variance analysis (ANOVA; *n* = 18). Assumptions of sphericity were verified with Mauchly’s test of sphericity (*P* ≤ 0.05). This analysis was followed by post hoc pairwise multiple comparisons with Tukey’s HSD test. If the assumptions of sphericity were violated, then a multivariate test was applied. If statistically significant differences were confirmed between the variables compared, but there was no significant interaction in the system of repeated measurements, planned comparison analysis was used. Then, both factors (incision closed with adhesive or surgical silk sutures) in subsequent weeks of the experiments were compared. However, statistical significance in hematological and biochemical indicators was tested with the Mann-Whitney test. Differences were noted as significant at *P* ≤ 0.05.

## Results

### Rearing indicators

Tagging did not influence fish behavior, activity, or feeding. No statistically significant differences were noted in the growth, condition, and FCR indexes analyzed in the tagged or control group fish from either of the experiments (*P* > 0.05; Table 4). No pikeperch mortality was noted in either of the experiments.

**Table 4:** Rearing indexes of pikeperch tagged with telemetry transmitters (experiment I—control group (SC) and silk suture group (ST) and experiment II—control group (GC) and adhesive suture group (GT)) on subsequent days of rearing (*d*_0_—initial day of rearing; *d*_7_, *d*_14_, *d*_21_, *d*_28_, *d*_35_—days 7, 14, 21, 28, and 35 of rearing, respectively) (mean ± SD, *n* = 18)

	Experiment I		Experiment II	
Index day of rearing	Group SC	Group ST	Group GC	Group GT
SL (cm)				
*d*_0_	16.89 (±0.52)	17.13 (±0.50)	19.57 (±0.73)	19.77 (±0.44)
*d*_35_	19.94 (±1.02)	19.57 (±1.37)	21.54 (±1.50)	21.29 (±1.59)
BW (g)				
*d*_0_	59.37 (±3.79)	60.19 (±5.29)	86.81 (±6.63)	86.99 (±6.41)
*d*_7_	66.97 (±5.52)	64.61 (±8.07)	93.91 (±10.56)	91.72 (±11.28)
*d*_14_	74.12 (±8.48)	69.57 (±12.15)	100.21 (±15.01)	96.61 (±16.83)
*d*_21_	82.69 (±9.13)	75.40 (±15.83)	107.04 (±20.06)	101.04 (±22.70)
*d*_28_	91.76 (±11.58)	83.28 (±20.42)	111.89 (±25.17)	105.46 (±27.76)
*d*_35_	100.19 (±15.48)	88.30 (±24.50)	118.44 (±30.27)	110.95 (±34.00)
DGR (g d^−1^)				
*d*_0_–*d*_7_	1.09 (±0.34)	0.63 (±0.67)	1.01 (±0.87)	0.67 (±0.98)
*d*_7_–*d*_14_	1.02 (±0.58)	0.71 (±0.70)	0.90 (±0.80)	0.70 (±0.94)
*d*_14_–*d*_21_	1.23 (±0.44)	0.83 (±0.67)	0.98 (±0.84)	0.63 (±0.88)
*d*_21_–*d*_28_	1.30 (±0.43)	1.13 (±1.35)	0.69 (±0.95)	0.63 (±0.82)
*d*_28_–*d*_35_	1.20 (±0.64)	0.72 (±0.65)	0.93 (±1.00)	0.78 (±0.98)
*d*_0_–*d*_35_	1.17 (±0.37)	0.80 (±0.62)	0.90 (±0.79)	0.68 (±0.89)
SGR (% d^−1^)				
*d*_0_–*d*_7_	1.70 (±0.48)	0.96 (±1.01)	1.07 (±0.94)	0.69 (±1.04)
*d*_7_–*d*_14_	1.40 (±0.84)	0.95 (±0.97)	0.85 (±0.81)	0.64 (±0.92)
*d*_14_–*d*_21_	1.57 (±0.66)	1.05 (±0.86)	0.84 (±0.74)	0.5 (±0.78)
*d*_21_–*d*_28_	1.46 (±0.40)	1.28 (±1.79)	0.51 (±0.81)	0.47 (±0.73)
*d*_28_–*d*_35_	1.19 (±0.63)	0.70 (±0.69)	0.68 (±0.85)	0.54 (±0.78)
*d*_0_–*d*_35_	1.46 (±0.38)	0.99 (±0.75)	0.79 (±0.73)	0.57 (±0.81)
*F* (−)				
*d*_0_	1.23 (±0.09)	1.20 (±0.09)	1.16 (±0.09)	1.12 (±0.06)
*d*_7_	1.25 (±0.08)	1.18 (±0.09)	1.15 (±0.10)	1.13 (±0.09)
*d*_14_	1.25 (±0.09)	1.18 (±0.10)	1.18 (±0.11)	1.13 (±0.10)
*d*_21_	1.26 (±0.08)	1.18 (±0.12)	1.17 (±0.11)	1.13 (±0.11)
*d*_28_	1.26 (±0.09)	1.21 (±0.22)	1.15 (±0.11)	1.12 (±0.13)
*d*_35_	1.25 (±0.09)	1.15 (±0.13)	1.16 (±0.12)	1.11 (±0.13)
FCR (−)				
*d*_0_–*d*_7_	0.70 (±0.03)	1.25 (±0.18)	1.16 (±0.33)	1.72 (±0.45)
*d*_7_–*d*_14_	0.93 (±0.16)	1.30 (±0.12)	1.55 (±0.5)	2.04 (±0.75)
*d*_14_–*d*_21_	0.86 (±0.13)	1.21 (±0.15)	1.55 (±0.51)	2.62 (±1.47)
*d*_21_–*d*_28_	0.89 (±0.04)	1.13 (±0.51)	2.89 (±1.93)	2.33 (±0.46)
*d*_28_–*d*_35_	1.08 (±0.11)	1.76 (±0.53)	1.91 (±0.92)	1.95 (±0.44)
*d*_0_–*d*_35_	0.88 (±0.04)	1.26 (±0.25)	1.62 (±0.49)	2.06 (±0.58)
Mortality (%)				
*d*_0_–*d*_35_	0	0	0	0

Details are provided in the “Materials and methods” section. No statistically significant differences between groups were noted within the same experiments (*P* > 0.05)

### Tagging effectiveness indicators

**Fig. 1:**
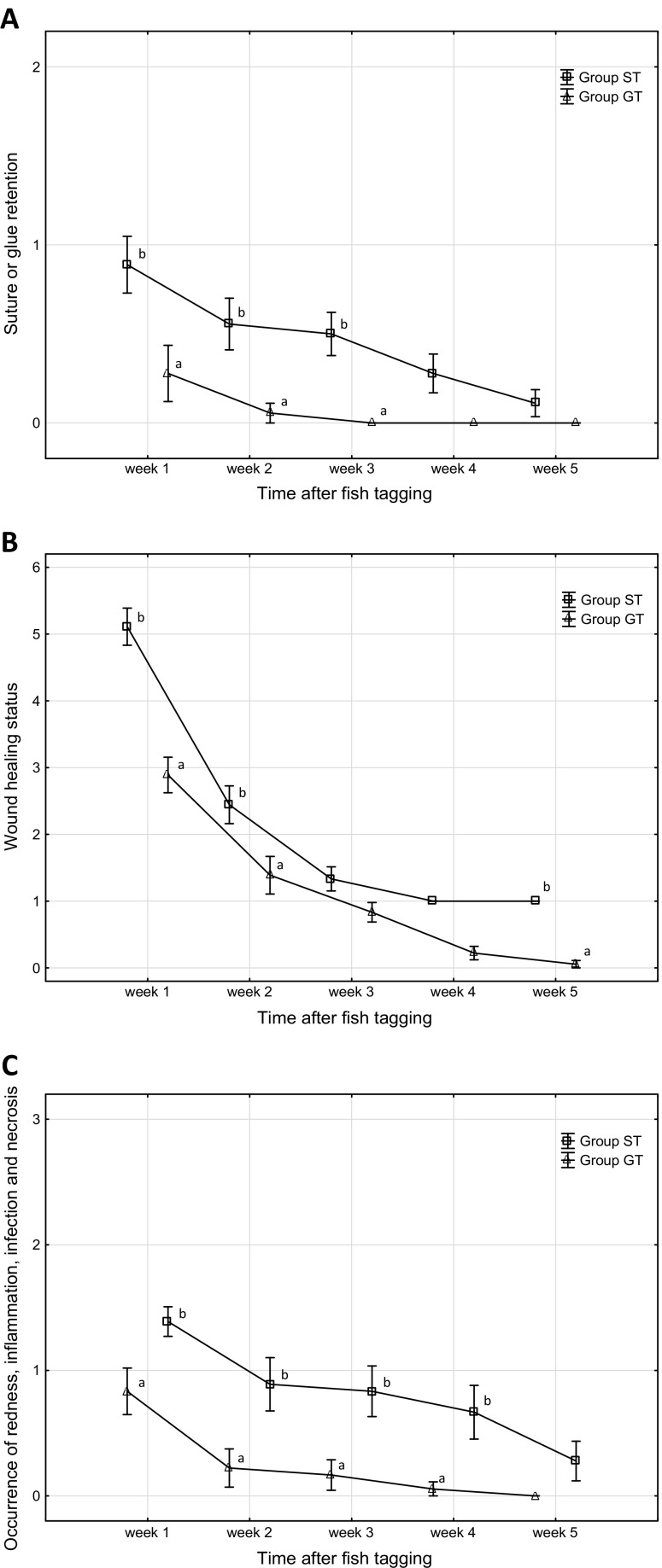
Wound healing and the state of silk sutures or tissue adhesive in two groups of pikeperch following the implantation of telemetry transmitters (group ST—fish in which wounds were closed with silk sutures; group GT—pikeperch in which wounds were closed with tissue adhesive). State of silk sutures or tissue adhesive (**a**; see Table 1). State of wound healing (**b**; see Table 2). Occurrence of redness, inflammation, and infection (**c**; see Table 3). Data with *different letter* indexes from the same week differ significantly statistically (*P* ≤ 0.05) (mean values ± SE)

The ranking values of silk and tissue adhesive suture-holding capacity in the tagged fish differed significantly statistically (Table 1 and Fig. 1a). The tissue adhesive in group GT was shed, in most cases, in the first week of the experiment. While in group ST, the silk sutures were shed throughout the experiment. The incision closure method applied was significant for the degree of holding capacity of the tissue adhesive or silk sutures that closed the incision (*P* < 0.05). At week 3 post-surgery, the tissue adhesive-holding capacity (rank) was still significantly lower than that of the silk sutures (Fig. 1a). In the group of fish with surgical silk sutures (ST), only one fish lost its TT tag during the experiment (in the second week; 94% retention), while in the group of fish in which tissue adhesive had been used (GT), TT tag retention was 100% for the duration of the experiment.

Implantation wounds healed faster in the fish from group GT, in which tissue adhesive had been used to close the incision (Table 2 and Fig. 1b). After the first week of rearing following tagging, the incisions of most of the fish from group GT were at level 3 in the range of incision index scores (*P* < 0.05; Fig. 1b). However, at the same time in the group of fish with silk sutures (ST), the implantation wound were at a mean level of 5 in the range of incision index scores and more than 50% of them were open. After the second week of rearing, the difference in healing rates decreased, but it remained statistically significant. The healing of the wounds of pikeperch from group GT oscillated between levels 1 and 2 in the range of incision index scores, while in group ST, it was between 2 and 3 (*P* < 0.05). After the third and fourth weeks, the differences between the two groups of tagged fish were no longer statistically significant. After the fifth week, the fish from group GT achieved level 0 in the range of incision index scores, while fish from group ST remained at level 1 (*P* < 0.05; Fig. 1b).

The method of incision closure had a statistically significant impact on the healing rate and the state of the wound (*P* < 0.05). In the groups of fish in which the implantation incision was closed with tissue adhesive, there were fewer specimens with redness at the incision site (GT—7, ST—11) or that exhibited evident symptoms of inflammation (GT—4, ST—7) (Fig. 1c). Additionally, the incisions of seven fish from this group (GT) were clean and were healing through primary adhesion (group ST—0 fish). No signs of inflammation were noted in specimens from group GT as early as week 4 of the experiment, while by week 5, there was a lack of redness. However, redness (1 fish) and inflammation (2 fish) were noted until the conclusion of the experiment in some specimens from group ST (Fig. 1c). None of the fish examined, regardless of the method used to close the incision, exhibited symptoms of infection or tissue necrosis. The methods used to close the incision permitted achieving uniform results (incision state) at the conclusion of the experiments. However, the closure of the incision following the implantation of transmitters using tissue adhesive was more advantageous. The analysis of the planned comparisons indicated that there were statistically significant differences in the assessments of incision inflammation until week 4 of the experiments (*P* < 0.05), and only in week 5 did these differences become statistically insignificant (*P* > 0.05).

### Hematological and biochemical indicators

Among the hematological indicators analyzed, statistically significant differences were only noted in experiment I (Table 5). Higher values of MCV and MCH were noted in the control group (*P* < 0.05), while intergroup differences in the values of the remaining indicators, i.e., WBC, RBC, HGB, HCT, MCHC, and PLT, were statistically insignificant (*P* > 0.05; Table 5).

**Table 5:** Hematological indexes of two pikeperch groups in which different implantation incision suturing was applied (experiment I—control group (SC) and silk suture group (ST) and experiment II—control group (GC) and adhesive suture group (GT)) (mean ± SD, *n* = 18)

		Experiment I		Experiment II	
		Group SC	Group ST	Group GC	Group GT
WBC	10^3^ μl^−1^	69.72 (±18.11)	71.14 (±12.74)	71.48 (±12.37)	71.73 (±16.93)
RBC	10^6^ μl^−1^	1.64 (±0.21)	1.63 (±0.21)	1.65 (±0.16)	1.55 (±0.23)
HGB	g l^−1^	36.61 (±4.75)	34.69 (±4.21)	38.67 (±4.49)	37.06 (±5.62)
HCT	%	27.14 (±3.07)	25.52 (±3.57)	26.69 (±2.86)	25.02 (±3.55)
MCV	fl	122.93 (±5.84) b	116.16 (±8.14) a	119.94 (±8.43)	120.08 (±8.52)
MCH	pg	22.26 (±1.39) b	21.26 (±1.14) a	23.34 (±1.63)	23.88 (±1.87)
MCHC	g l^−1^	181.50 (±8.89)	183.50 (±8.45)	195.22 (±8.03)	199.35 (±10.65)
PLT	10^3^ μl^−1^	18.89 (±4.87)	20.25 (±15.49)	20.33 (±5.88)	19.06 (±7.75)

Details are provided in the “Materials and methods” section. Groups with different letter indexes from the same experiment differ significantly statistically (*P* ≤ 0.05)

The results of the biochemical determinations in experiment I indicated that the pikeperch from the control group had a statistically significant, threefold higher level of CREA, a higher level of magnesium (Mg), and a lower level of ammonia (NH_3_) (*P* < 0.05; Table 6). The remaining parameters in both experiments did not differ significantly statistically (*P* > 0.05) with the exception of ALP, the activity of which was lower in the TT tagged fish in both experiments (*P* < 0.05; Table 6).

**Table 6:** Biochemical indexes of two pikeperch groups in which different implantation incision suturing was applied (experiment I—control group (SC) and silk suture group (ST) and experiment II—control group (GC) and adhesive suture group (GT) (mean ± SD, *n* = 18)

		Experiment I		Experiment II	
		Group SC	Group ST	Group GC	Group GT
CREA	mg dl^−1^	0.27 (±0.19) b	0.09 (±0.07) a	0.24 (±0.20)	0.22 (±0.21)
TP	g dl^−1^	4.11 (±0.36)	4.01 (±0.35)	3.73 (±0.36)	3.63 (±0.43)
BIL-T	mg dl^−1^	0.07 (±0.08)	0.06 (±0.03)	0.13 (±0.13)	0.12 (±0.16)
ALT	U l^−1^	59.83 (±54.85)	44.29 (±42.27)	43.56 (±45.09)	64.88 (±66.89)
ALP	U l^−1^	74.06 (±17.71) b	54.94 (±21.59) a	70.44 (±17.88) b	54.24 (±14.73) a
Ca	mg dl^−1^	10.58 (±1.83)	10.44 (±0.93)	10.83 (±0.50)	10.63 (±0.44)
ALB	g dl^−1^	1.46 (±0.23)	1.48 (±0.13)	1.31 (±0.23)	1.26 (±0.23)
GLOB	g dl^−1^	2.65 (±0.20)	2.52 (±0.28)	2.42 (±0.20)	2.36 (±0.28)
GLU	mg dl^−1^	65.00 (±11.66)	55.35 (±14.21)	64.83 (±26.58)	66.06 (±28.7)
Mg	mg dl^−1^	2.57 (±0.17) b	2.43 (±0.14) a	2.51 (±0.11)	2.48 (±0.08)
NH_3_	μg dl^−1^	670.32 (±176.25) a	899.55 (±398.36) b	477.91 (±171.92)	464.81 (±133.57)

Details are provided in the “Materials and methods” section. Groups with different letter indexes from the same experiment differ significantly statistically (*P* ≤ 0.05)

## Discussion

Tagging pikeperch with TT was not confirmed to impact growth in either of the experiments. Similar effects from telemetry transmitter implantation were observed in green sturgeon (*Acipenser medirostris*) (TL 45.4 ± 0.6 cm), in which, after 140 days of the experiment, telemetry transmitter tagging was not found to have had any impact on growth indexes (Miller et al. 2014). The telemetry transmitter implantation in gilthead bream (*Sparus aurata*) (BW 289 ± 53 g) had no significant impact on the growth or behavioral reactions (feed ingestion or physical activity) of the tagged fish. Although immediately following tagging slight differences in mobility were noted, after a few days, the physical activity of the fish returned to that observed prior to the implantation procedure (Montoya et al. 2012). Different observations are reported for brown trout (*Salmo trutta*), among other species. Implanting wild brown trout (TL 19.4 ± 5.2 cm) with telemetry transmitters had a negative impact on their growth in natural conditions. The slowed growth rates in this species were explained by the higher-energy requirements for incision healing and by the altered physical activity of the fish (Jepsen et al. 2008). Slower growth 144 days following the implantation procedure was also reported in bluefish (*Pomatomus saltatrix*), a marine fish of the order Perciformes (Thorstad et al. 2009). Although information can be found in the literature regarding the negative impact of telemetry transmitter tagging on the growth parameters of some fish species, most scientists investigating this issue do not report decreases in the values of these indexes (Adams et al. 1998; Martinelli et al. 1998; Bégout-Anras et al. 2003; Cooke et al. 2003; the current study). Presumably, the root of these differences stem mainly from species differences, size differences, the origins of the tagged fish, and various implantation techniques. The conditions under which the studies are conducted, i.e., in a laboratory/hatchery or in the wild, could also be of significance.

Among the hematological indexes determined, statistically significant differences were only noted in the experiment in which the pikeperch implantation incision was closed with silk sutures. The fish from group ST had lower MCV and MCH values in the systemic blood (*P* < 0.05). The decreased values of these parameters can indicate lowered iron levels and water-electrolyte disruptions caused by blood loss and chronic inflammation of the implantation incision in group ST. Neves et al. (2016) report similar views. Most of the biochemical parameters determined in the blood of juvenile pikeperch following the implantation of telemetry transmitters did not differ significantly from the values of these parameters in the fish from the control groups. Very similar results were reported for gilt-head bream, which, after being tagged with TT, just like with pikeperch (experiments I and II), most parameters were comparable to those of the control group, for example, blood glucose levels (Montoya et al. 2012). In their study of bighead carp (*Hypophthalmichthys nobilis*), Luo et al. (2014) observed significant increases in the levels of blood glucose and cortisol 3 h following implantation in both the group of fish tagged with TT and in that that was subjected to the surgical procedure without implantation (sham sample). Increases in these parameters are evidence of the stress induced in fish by implantation. However, the effect was short term, because after 24 h, both of these parameters returned to initial values. The results reported by Luo et al. (2014) indicate that the main stress factor for the fish is the abdominal incision and not TT implantation. Similarly, largemouth bass (*Micropterus salmoides*), which spent about a year (362 days) in open waters after being tagged with TT, did not exhibit significant differences in glucose levels or in total protein and magnesium levels, which reflect nutritional status (Caputo et al. 2009). In turn, pikeperch levels of magnesium were comparable with those of the control group only in experiment II. It is also noteworthy that in the pikeperch from experiments I and II, the values of the two other nutritional status indexes (total protein and calcium) also did not differ significantly from those in the fish from the control group. Thus, it can be concluded that the TT implantation procedure did not lead to changes in the nutritional status of the pikeperch. In group ST (experiment I), significant decreases in the levels of creatinine in the serum and increased ammonia concentrations (134% of the values determined for group SK) were noted. The values of these indexes, which are markers of kidney function and metabolism, among other factors, are evidence of intense protein catabolism associated with increased energy demands when there is inflammation and incision healing (Prakash et al. 2014). The differences referred to above observed in experiment I (silk sutures) were not noted in the pikeperch from experiment II, in which the implantation incision closed with tissue adhesive healed more quickly. Alkaline phosphatase activity is a helpful marker of the health status of bodies. Lowered levels of the activity of this enzyme in both groups (ST and GT) indicate disruption in liver function associated with the synthesis of factors responsible for hemostasis, increased protein catabolism, and incision healing. Decreases in ALP activity can stem directly from a decelerated glycogen synthesis rate, malnutrition, or the wasting/exhaustion of the body (Shaffi 1979).

Wound healing following the surgical implantation of transmitters has a significant impact on fish welfare and the success of telemetry studies (Rub et al. 2014). The telemetry transmitter implantation wounds healed faster in the group of juvenile pikeperch, in which the incision was closed with tissue adhesive. The layer of adhesive peeled off after the first week as soon as the opposing edges of the incision had joined, and the state of healing was satisfactory 2 weeks after tag implantation. Different results were reported in eel (*Anguilla anguilla*) (TL 56.2–78.0 cm) following TT implantation in the body cavity (tag weight <0.6% BW). The incisions healed more quickly in the group of fish in which closure was done with silk sutures (40 days—time between implantation and complete incision healing) and not with tissue adhesive (52 days). However, the silk sutures did cause inflammation, and they contributed to higher mortality rates in the tagged ell (silk sutures—60%; tissue adhesive—10%, incision not closed—20%). Significantly, the implantation incisions that were not closed healed most quickly (28 days) (Baras and Jeandrain 1998). In our study, after the first week of the experiment, 61% of the pikeperch in which the implantation incision was closed with sutures exhibited redness, while in 39%, there was distinct inflammation. In group GT, these symptoms were noted in 39 and 22% of specimens, respectively. After the conclusion of the experiment (5 weeks), no specimens from group GT exhibited these symptoms, while in group ST, 16.7% of the fish still did. In this group, redness/inflammation was noted mainly in the vicinity of the silk sutures that closed the incision. Incision healing in pikeperch specimens in which the sutures fell out earlier (1–2 weeks following tagging) was faster, and inflammation did not occur. In largemouth bass, inflammation, infection, and even tissue necrosis in the vicinity of the sutures following telemetry transmitter implantation were confirmed in 7 of 17 tagged fish (41%), despite the application of absorbable monofilament silk and the fact that 362 days had passed since the implantation procedure had been performed (Caputo et al. 2009).

Brown trout that was tagged with TT was confirmed to have symptoms of inflammation and tissue necrosis at the antenna exit site on the body (Jepsen et al. 2008). Bauer et al. (2005) observed that this site could even become infected. However, beside some slight ecchymosis of the skin near the antenna, no other symptoms of infection or inflammation were noted in pikeperch. These relatively slight changes resulted from other specimens taking hold of the antennae (Z. Zakęś, personal observation). It is possible that in the wild, this phenomenon would not occur or that it might occur less frequently than in the limited space of rearing tanks.

The expulsion of surgically implanted TT tagging is a phenomenon that is described in the literature (Burrell et al. 2000; Jepsen et al. 2002). It occurs most frequently within the first few weeks after tagging and depends on many factors (species, fish health status, tag weight/size, and environmental conditions) (Jepsen et al. 2002). Fish shed tags in the following three ways: through the implantation incision, through the skin, or through the intestines (which is a rare occurrence) (Jepsen et al. 2002). During the experiment, among the 18 fish tagged, only 1 specimen shed its tag. The fish shed its tag in the second week of rearing through the implantation incision that had been closed with silk sutures (group ST).

## Summary

The surgical method for tagging pikeperch with telemetry transmitters did not have a negative impact on either behavior or physiological processes. Tissue adhesive was better for suturing implantation incisions as fewer statistically significant differences were noted in the hematological and biochemical parameters of fish in which this method was applied. Significantly, this method promoted faster healing of the implantation incision. Additionally, closing the incision with adhesive, in contrast to silk sutures, did not result in inflammation or infection. In consideration of the preceding arguments, using tissue adhesive to close implantation incisions could be recommended as a method for tagging juvenile pikeperch with TT. It is crucial, however, to select the appropriate size and weight of transmitter and to use the appropriate implantation techniques (by trained personnel) and surgical equipment (Mulcahy 2003).

## References

[CR1] Adams NS, Rondorf DW, Evans SD, Kelly JE, Perry RW (1998). Effects of surgically and gastrically implanted radio transmitters on swimming performance and predator avoidance of juvenile Chinook salmon (*Oncorhynchus tshawytscha*). Can J Fish Aquat Sci.

[CR2] Baras E, Lagardère JP (1995). Fish telemetry in aquaculture: review and perspectives. Aquacult Int.

[CR3] Baras E, Jeandrain D (1998). Evaluation of surgery procedures for tagging eel *Anguilla anguilla* with telemetry transmitters. Hydrobiologia.

[CR4] Bartley DM, Rana K (1998). Stocking inland waters of the Islamic Republic of Iran. The FAO Aquaculture Newsletter.

[CR5] Bauer C, Unfer G, Loupal G (2005). Potential problems with external trailing antennas: antenna migration and ingrowth of epithelial tissue—a case study from a recaptured nase, *Chondrostoma nasus* (L.). J Fish Biol.

[CR6] Bégout-Anras ML, Covés D, Dutto G, Laffargue P, Lagardére F (2003). Tagging juvenile seabass and sole with telemetry transmitters: medium term effects on growth. ICES J Mar Sci.

[CR7] Bridger CJ, Booth RK (2003). The effects of biotelemetry transmitter presence and attachment procedures on fish physiology and behavior. Rev Fish Sci.

[CR8] Brinn RP, Marcon JL, Gomes DM, Abreu LC, Baldisseroto B (2012). Stress responses of the endemic freshwater cururu stingray (*Potamotrygon cf. histrix*) during transportation in the Amazon region of the Rio Negro. Comp Biochem Physiol A.

[CR9] Burrell KH, Isely JJ, Bunnell DB, Lear DH, Dolloff CA (2000). Seasonal movement of Brown trout in a southern Appalachian River. Trans Am Fish Soc.

[CR10] Caputo M, O’Connor CM, Hasler CT, Hanson KC, Cooke SJ (2009). Long-term effects of surgically implanted telemetry tags on the nutritional physiology and condition of wild freshwater fish. Dis Aquat Org.

[CR11] Clauss TM, Dove A, Arnold J (2008). Hematologic disorders of fish. Vet Clin Exot Anim.

[CR12] Cooke SJ, Graeb BDS, Suski CD, Ostrand KG (2003). Effects of suture material on incision healing, growth and survival of juvenile largemouth bass implanted with miniature radio transmitters: case study of a novice and experienced fish surgeon. J Fish Biol.

[CR13] Cooke SJ, Midwood JD, Thiem JD, Klimley P, Lucas MC, Thorstad EB, Eiler J, Holbrook C, Ebner BC (2013). Tracking animals in freshwater with electronic tags: past, present and future. Anim Biotelem.

[CR14] Cooke SJ, Woodley CM, Eppard MB, Brown RS, Nielsen JL (2011). Advancing the surgical implantation of electronic tags in fish: a gap analysis and research agenda based on a review of trends in intracoelomic tagging effects studies. Rev Fish Biol Fish.

[CR15] Deters KA, Brown RS, Carter KM, Boyd JW, Eppard MB, Seaburg AG (2010). Performance assessment of suture type, water temperature, and surgeon skill in juvenile Chinook salmon surgically implanted with acoustic transmitters. Trans Am Fish Soc.

[CR16] Dudgeon CL, Pollock KH, Braccini JM, Semmens JM, Barnett A (2015) Integrating acoustic telemetry into mark–recapture models to improve the precision of apparent survival and abundance estimates. 178:761–772. doi: 10.1007/s00442-015-3280-z 10.1007/s00442-015-3280-z 25740335

[CR17] FAO (2012) Cultured Aquatic Species Information Programme—*Sander lucioperca* (Linnaeus, 1758). http://www.fao.org/fishery/culturedspecies/Sander_lucioperca/en. Accessed 25 October 2016

[CR18] FAO (2016) Global capture production 1950–2014. http://www.fao.org/fishery/statistics/global-capture-production/query/en. Accessed 22 October 2016

[CR19] Hansson S, Arrhenius F, Nellbring S (1997). Benefits from fish stocking—experiences from stocking young-of-the-year pikeperch, *Stizostedion lucioperca* L. to a bay in the Baltic Sea. Fish Res.

[CR20] Horký P, Slavík O, Bartoš L, Kolářová J, Randák T (2006). The effect of the moon phase and seasonality on the behavior of pikeperch in the Elbe River. Folia Zool.

[CR21] Jepsen N, Koed A, Thorstad EB, Baras E (2002). Surgical implantation of telemetry transmitters in fish: how much have we learned?. Hydrobiologia.

[CR22] Jepsen N, Mikkelsen JS, Koed A (2008). Effects of tag and suture type on survival and growth of brown trout with surgically implanted telemetry tags in the wild. J Fish Biol.

[CR23] Jepsen N, Pedersen S, Thorstad E (2000). Behavioural interactions between prey (trout smolts) and predators (pike and pikeperch) in an impounded river. Regulated Rivers: Res Mgmt.

[CR24] Koed A, Mejlhede P, Balleby K, Aarestrup K (2000). Annual movement and migration of adult pikeperch in a lowland river. J Fish Biol.

[CR25] Koed A, Balleby K, Mejlhede P (2002). Migratory behaviour of adult pikeperch (*Stizostedion lucioperca*) in a lowland river. Hydrobiologia.

[CR26] Kristan J, Stara A, Polgesek M, Drasovean A, Kolarova J, Priborsky J, Blecha M, Svacina P, Policar T, Velisek J (2014). Efficacy of different anaesthetics for pikeperch (*Sander lucioperca* L.) in relation to water temperature. Neuroendocrinol Lett.

[CR27] Lambooij B, Pilarczyk M, Białowąs H, Reimert H, André G, van de Vis H (2009). Anaesthetic properties of Propiscin (Etomidaat) and 2-phenoxyethanol in the common carp (*Cyprinus carpio* L.), neural and behavioural measures. Aquac Res.

[CR28] Luo H, Duan X, Liu S, Chen D (2014). Effects of surgically implanted dummy ultrasonic transmitters on physiological response of bighead carp *Hypophthalmichthys nobilis*. Fish Physiol Biochem.

[CR29] Martinelli TL, Hansel HC, Shively RS (1998). Growth and physiological responses to surgical and gastric radio-transmitter implantation techniques in subyearling Chinook salmon. Hydrobiologia.

[CR30] Miller EA, Froehlich HE, Cocherell DE, Thomas MJ, Cech JJ, Klimley AP, Fangue NA (2014). Effects of acoustic tagging on juvenile green sturgeon incision healing, swimming performance, and growth. Environ Biol Fish.

[CR31] Montoya A, López-Olmeda JF, Lopez-Capel A, Sánchez-Vázquez FJ, Pérez-Ruzafa A (2012). Impact of a telemetry-transmitter implant on daily behavioral rhythms and physiological stress indicators in gilthead seabream (*Sparus aurata*). Mar Environ Res.

[CR32] Mulcahy DM (2003). Surgical implantation of transmitters into fish. ILAR J.

[CR33] Neves JV, Caldas C, Ramos MF, Rodrigues PNS (2016). Hepcidin-dependent regulation of erythropoiesis during anemia in a teleost fish, *Dicentrarchus labrax*. PLoS One.

[CR34] Pereira L, Fernandes MN, Martinez CBR (2013). Hematological and biochemical alterations in the fish *Prochilodus lineatus* caused by the herbicide clomazone. Environ Toxicol Pharmacol.

[CR35] Prakash MM, Gaherwal S, Soni R, Wast N (2014). Cypermethrin induced biochemical changes in kidney of *Clarias batrachus*. World Appl Sci J.

[CR36] Rub AMW, Jepsen N, Liedtke TL, Moser ML, Weber ES (2014). Surgical insertion of transmitters and telemetry methods in fisheries research. Am J Vet Res.

[CR37] Saura A (1996). Use of hot branding in marking juvenile pikeperch (*Stizostedion lucioperca*). Ann Zool Fenn.

[CR38] Shaffi SA (1979). Effects of starvation on tissue and serum gluconeogenic enzymes, alkaline phosphatase and tissue glycogen in the fresh water catfish, *Heteropneustes fossilis* (Bloch). Acta Physiol Acad Sci Hung.

[CR39] Steenfeldt S, Fontanie P, Overton JL, Policar T, Toner D, Falahatkar B, Horváth Á, Khemis IB, Hamza N, Mhetli M (2015) Current status of Eurasian percid fishes aquaculture. In: Kestemont P, Dabrowski K, Summerfelt RC (ed) Biology and culture of percid fishes—principles and practices. Springer Dordrecht, pp. 817–842.

[CR40] Szkudlarek M, Zakęś Z (2007). Effect of stoking density on survival and growth performance of pikeperch, *Sander lucioperca* (L.), larvae under controlled conditions. Aquacult Int.

[CR41] Thorstad EB, Kerwath SE, Attwood CG, Økland F, Wilke CG, Cowley PD, Næsje TF (2009). Long-term effects of two sizes of surgically implanted acoustic transmitters on a predatory marine fish (*Pomatomus saltatrix*). Mar Freshw Res.

[CR42] Thorstad EB, Rikardsen AH, Alp A, Økland F (2013). The use of electronic tags in fish research—an overview of fish telemetry methods. Turk J Fish Aquat Sci.

[CR43] Val AL, Affonso EG, Dealmeidaval VMF (1992). Adaptive features of amazon fishes—blood characteristics of curimata (*Prochilodus* cf. *nigricans*, osteichthyes). Physiol Zool.

[CR44] Vehanen T, Lahti M (2003). Movements and habitat use by pikeperch (*Stizostedion lucioperca* (L.)) in a hydropeaking reservoir. Ecol Freshw Fish.

[CR45] Wagner GN, Cooke SJ, Brown RS, Deters KA (2011). Surgical implantation techniques for electronic tags in fish. Rev Fish Biol Fish.

[CR46] Winter JD, Murphy BR, Willis DW (1996). Advances in underwater biotelemetry. Fisheries techniques.

[CR47] Zakęś Z (2007). Out-of-season spawning of cultured pikeperch (*Sander lucioperca* (L.)). Aquac Res.

[CR48] Zakęś Z, Hopko M (2013). Tagging juvenile pikeperch (*Sander lucioperca* (L.)) in the cheek with passive integrated transponders (PIT)—impact on rearing indexes and tag retention. Arch Pol Fish.

[CR49] Zakęś Z, Kapusta A, Hopko M, Szczepkowski M, Kowalska A (2015). Growth, survival and tag retention in juvenile pikeperch (*Sander lucioperca*) in laboratory conditions. Aquac Res.

[CR50] Zorriehzahra MJ, Hassan MD, Gholizadeh M, Saidi AA (2010). Study of some hematological and biochemical parameters of rainbow trout (*Oncorhynchus mykiss*) fry in western part of Mazandaran province, Iran. Iran J Fish Sci.

